# Aesthetic Medicine in Moroccan Dental Practice: A Cross-Sectional Survey of Knowledge, Attitudes, and Implementation

**DOI:** 10.7759/cureus.91890

**Published:** 2025-09-09

**Authors:** Loubna Amminou, Sara Boukssim, Saliha Chbicheb

**Affiliations:** 1 Department of Oral Surgery, Faculty of Dental Medicine, Mohammed V University, Rabat, MAR

**Keywords:** aesthetic medicine, botulinum toxin, cross-sectional survey, dental practice, dermal fillers

## Abstract

Objectives: This study aims to explore the extent to which aesthetic medical procedures, such as botulinum toxin and dermal fillers, are integrated into dental practice by Moroccan dentists, and to assess their knowledge, attitudes, and perceived legal framework regarding these interventions.

Materials and methods: A structured eight-item questionnaire, developed via Google Forms (Google Inc., Mountain View, CA, USA), was distributed over one month through professional Moroccan dental groups on social media platforms and via direct online outreach. The survey targeted licensed dentists across different age groups and regions of Morocco.

Results: A total of 166 dentists responded to the survey. Of these, 65.1% (n = 108) were female and 34.9% (n = 58) were male. Most respondents (61.4%, n = 102) were between 27 and 35 years old. Only 11.4% (n = 19) of dentists reported actively performing aesthetic procedures in their daily practice. However, 33.1% (n = 55) stated that they regularly receive patient inquiries about aesthetic treatments. In contrast, 50% (n = 83) believed that dentists should be authorized to perform aesthetic medical procedures. In contrast, only 23.5% (n = 39) reported being aware of the Moroccan legal framework surrounding these practices.

Conclusions: Aesthetic procedures are still rarely practiced by Moroccan dentists, despite growing patient interest. Limited legal awareness and training may explain the gap between demand and implementation.

## Introduction

In recent years, there has been a notable rise in the use of dermal fillers, lasers, and lifting threads as part of minimally invasive aesthetic procedures [1,2]. These techniques, once the exclusive domain of plastic surgeons and dermatologists, are now being adopted by a broader range of healthcare professionals, including dentists.

Dentists are especially well-suited to address interventions in the perioral region, due to their comprehensive understanding of craniofacial muscles, bone structures, and neurosensory anatomy. This makes them ideal candidates for delivering safe, anatomically informed aesthetic treatments [3,4]. Botox and dermal fillers have demonstrated both cosmetic and therapeutic value in managing conditions such as gummy smile, temporomandibular joint disorders, bruxism, and masseter hypertrophy [1]. Internationally, the incorporation of aesthetic medicine into dental practice is gaining legitimacy. In the United Kingdom, nearly one-quarter of cosmetic injectable treatments, such as Botox and dermal fillers, are administered by dentists, highlighting the expansion of professional roles beyond traditional boundaries [5]. Morocco is no exception. An increasing number of Moroccan dentists are turning to aesthetic medicine, driven not only by the appeal of these procedures but also by growing patient demand.

Despite the increasing global interest in facial aesthetic procedures among dental professionals, there is a lack of empirical data on how these practices are perceived and implemented in Morocco. While dentists in many countries are integrating botulinum toxin (BoNT-A), dermal fillers, and lifting threads into their clinical practice, both for cosmetic and therapeutic purposes, the regulatory framework, training opportunities, and clinical adoption in Morocco remain largely undefined. Most existing studies focus on European or North American contexts, overlooking regional variations in professional readiness, legal constraints, and educational infrastructure.

This study seeks to address this gap by evaluating the current perception, level of training, and clinical practice of aesthetic medicine among Moroccan dentists, with a particular focus on procedures involving Botox, dermal fillers, and lifting threads in the perioral region. By capturing qualitative data on dentists' attitudes, experiences, and barriers to practice, this research aims to provide a foundational understanding of how aesthetic medicine is evolving in Moroccan dental practice. The findings are intended to inform policymakers, dental educators, and regulatory bodies and to support the development of standardized training programs and legal guidelines that ensure patient safety and professional competence in this emerging field.

## Materials and methods

Study design and setting

This study was conducted as a descriptive, cross-sectional survey carried out over one month in January 2025. Data were collected anonymously through a structured online questionnaire developed and distributed by the research team.

Population and sampling technique

The target population consisted of approximately 6,000 licensed dentists currently practicing in Morocco. The survey targeted dentists practicing in private clinics, since these settings represent the primary context in which aesthetic medical procedures are offered in Morocco. Using Epi Info™ software (Centers for Disease Control and Prevention, Atlanta, GA, USA), the minimum required sample size was calculated based on a 50% expected frequency, a 5% margin of error, and an 80% confidence level. The resulting minimum sample size was 160 participants. A convenience sampling method was applied, where participation was voluntary and dependent on respondents’ accessibility and willingness to complete the questionnaire.

Inclusion and exclusion criteria

Dentists were eligible to participate if they held a valid dental license and were actively practicing in Morocco at the time of the study and provided informed consent by voluntarily completing the questionnaire. Dentists were excluded if they were retired, on academic leave, or not currently practicing, and if they did not complete the questionnaire in full.

Data collection instrument

The authors designed an original questionnaire in French after a review of related literature and current dental practices. The questionnaire included eight closed-ended questions, divided into five thematic sections: demographic information (age, gender, and region of practice); clinical practice related to aesthetic medicine (types of procedures performed, frequency, and patient demand); motivations and barriers influencing engagement in aesthetic medicine; opinions on the legitimacy of aesthetic procedures within the dental profession; and knowledge of national legislation governing dental practice in aesthetic medicine.

The questionnaire was self-administered online using Google Forms (Google Inc., Mountain View, CA, USA) and disseminated via professional dental groups on social media platforms as well as direct contact through professional networks (Appendices). The survey took approximately three to five minutes to complete. No personally identifying information was collected.

Ethical considerations

Ethical approval for this study was obtained from the Ethics Committee for Biomedical Research of Mohammed V University in Rabat (IORG0006594), under the approval number CERB 56-25. All participants were informed of the study’s objectives before starting the survey. Participation was entirely voluntary, and informed consent was implied upon completion of the form. The study complied with the principles outlined in the Declaration of Helsinki, ensuring the confidentiality and autonomy of all participants.

Statistical analysis

Data were entered into Microsoft Excel 2019 (Microsoft Corp., Redmond, WA, USA) for cleaning and coding, and all statistical analyses were performed using Jamovi (The Jamovi Project, Sydney, Australia) version 2.6.23. Descriptive statistics were calculated for all variables. Categorical variables were summarized as frequencies and percentages. Associations between categorical variables were assessed using Pearson’s Chi-square (χ²) test when all expected cell counts were ≥5. For contingency tables with expected counts <5, Fisher’s exact test was used to ensure accuracy. A two-tailed p-value <0.05 was considered statistically significant.

In the bivariate analysis, the following associations were examined: age group × performing aesthetic medicine procedures, gender × performing aesthetic medicine procedures, receiving patient requests × performing aesthetic medicine procedures, and performing aesthetic medicine procedures × knowledge of Moroccan legislation. Results are presented in contingency tables showing the observed frequencies and row percentages for each category, along with test statistics, degrees of freedom, and p-values.

## Results

Background information of participants

A total of 166 dentists participated in the study. Among them, 65.1% (n = 108) were female and 34.9% (n = 58) were male. The majority of respondents (61.4%, n = 102) were young dentists aged between 27 and 35 years, while 25.9% (n = 43) were aged between 35 and 50, and 12.7% (n = 21) were above 50.

Geographically, participants were distributed as follows: Rabat-Salé-Zemmour-Zaer (30.7%, n = 51), Marrakech-Safi (24.7%, n = 41), Casablanca-Settat (20.5%, n = 34), Tanger-Tétouan-Hoceima (13.3%, n = 22), Fès-Boulemane (3.6%, n = 6), and other regions (7.2%, n = 12). Regarding aesthetic medicine, only 11.4% (n = 19) of dentists reported performing aesthetic procedures in their daily practice. However, 33.1% (n = 55) of the respondents stated that they receive requests from patients for aesthetic treatments.

Among those not practicing aesthetic medicine (n = 147), 59.2% (n = 87) prefer to limit their practice to dental medicine; 21.8% (n = 32) plan to incorporate aesthetic procedures after receiving proper training; 13.6% (n = 20) cited a lack of training as the reason; and 5.4% (n = 8) indicated they have not received any patient requests for such procedures. Half of the respondents (50%, n = 83) believe that dentists should be allowed to perform aesthetic medical procedures. In contrast, only 23.5% (n = 39) reported having any knowledge of the Moroccan legislation governing the practice of aesthetic medicine by dentists (Tables 1-2).

**Table 1:** Sociodemographic characteristics

Characteristics	N (166)	% of total
Gender		
Female	108	65.1%
Male	58	34.9%
Age		
27-35	102	61.4%
35-50	43	25.9%
>50	21	12.7%
Region		
Casablanca-Settat	34	20.5%
Fes-Bouleman	6	3.6%
Marrakech-Safi	41	24.7%
Rabat-Sale-Zemmour-Zaer	51	30.7%
Tanger-Tetouan-Houceima	22	13.3%
Other regions	12	7.2%

**Table 2:** Clinical practice related to aesthetic medicine

Clinical practice related to aesthetic medicine	N (166)	% of total
Do you receive requests from your patients for aesthetic medicine?	55	33.1%
In your daily practice, do you perform aesthetic medicine procedures?	19	11.4%
Why don’t you practice aesthetic medicine in your clinic? (among respondents not currently practicing aesthetic medicine, n = 147)	n = 147	
I am not trained	20	13.6%
I plan to do it after receiving training	32	21.8%
I prefer to limit myself to the practice of dental medicine	87	59.2%
I have no request	8	5.4%
Should dentists perform aesthetic medicine?	83	50%
Do you have any knowledge of Moroccan legislation regarding the practice of aesthetic medicine?	39	23.5%

Association between age group and performing aesthetic medicine procedures

No statistically significant association was observed between age group and the performance of aesthetic medicine procedures (Fisher's exact test, p = 0.88). The proportion of dentists performing these procedures was relatively similar across age groups: 10.8% in the 27-35-year group, 14.0% in the 35-50-year group, and 9.5% among those over 50 years old (Table 3).

**Table 3:** Association between age group and performing aesthetic medicine procedures Percentages are based on line totals. Fisher’s exact Test, p = 0.880.

Age group	No	Yes	Total n (%)
27-35	91 (89.2%)	11 (10.8%)	102 (100%)
35-50	37 (86.0%)	6 (14.0%)	43 (100%)
>50	19 (90.5%)	2 (9.5%)	21 (100%)
Total	147 (88.6%)	19 (11.4%)	166 (100%)

Similarly, there was no statistically significant association between gender and performing aesthetic medicine procedures (χ²(1) = 2.141, p = 0.143). Although a higher proportion of male dentists (17.2%) reported performing aesthetic procedures compared with female dentists (8.3%), this difference was not statistically significant (Table 4).

**Table 4:** Association between gender and performing aesthetic medicine procedures Percentages are based on line totals. χ²(1) = 2.95, p = 0.086.

Gender	No	Yes	Total n (%)
Female	99 (91.7%)	9 (8.3%)	108 (100%)
Male	48 (82.8%)	10 (17.2%)	58 (100%)
Total	147 (88.6%)	19 (11.4%)	166 (100%)

A statistically significant association was found between receiving patient requests for aesthetic medicine and actually performing these procedures (χ²(1) = 39.960, p < 0.001). Among dentists who reported receiving such requests, 34.5% performed aesthetic medicine procedures, compared with none among those who did not receive requests (Table 5).

**Table 5:** Association between patient request and performing aesthetic medicine procedures Percentages are based on line totals. χ²(1) = 39.960, p < 0.001.

	Performing procedures		
Patient request	No	Yes	Total n (%)
No request	111 (100.0%)	0 (0.0%)	111 (100%)
Request	36 (65.5%)	19 (34.5%)	55 (100%)
Total	147 (88.6%)	19 (11.4%)	166 (100%)

A statistically significant association was observed between performing aesthetic medicine procedures and knowledge of Moroccan legislation governing such practices (Fisher’s exact test, p < 0.001). Dentists who performed aesthetic procedures were more likely to report knowledge of relevant legislation (73.7%) compared with those who did not perform these procedures (17%) (Table 6).

**Table 6:** Association between performing aesthetic medicine and knowledge of Moroccan legislation Percentages are based on line totals. Fisher’s Exact Test, p < 0.001.

	Knowledge of Moroccan legislation		
Performing procedures	No	Yes	Total n (%)
No	122 (83%)	25 (17%)	147 (100%)
Yes	5 (26.3%)	14 (73.7%)	19 (100%)
Total	127 (76.5%)	39 (23.5%)	166 (100%)

## Discussion

In recent years, aesthetic medicine has evolved toward minimally invasive approaches and increased accessibility, with a growing uptake among both younger adults and older patients [6]. Within this landscape, dentists occupy a unique position at the medical-esthetic interface [2]. Consistent with prior clinical reviews, our findings support the role of BoNT-A and hyaluronic acid (HA) fillers as adjuncts to orofacial harmony and dental rehabilitation, particularly in indications such as hyperfunctional perioral muscles (gummy smile, perioral rhytids, oral commissure ptosis, and chin dimpling) [4,7-9]. Our results also echo evidence that injections are used therapeutically for bruxism, masseter hypertrophy, and chronic orofacial pain [1].

In this study, 33.1% of dentists reported patient requests, similar to Osailan et al.'s survey [10], yet only 11.4% currently practice, lower than Zargaran et al.'s survey [5] (24%), likely due to limited training opportunities and unclear regulation. While 22.3% would consider practicing after training, 58.8% preferred to remain within conventional dentistry. In Riyadh, Al Hamdan et al. [11] also found very low practice rates (1.2% for BoNT-A and 0.9% for dermal fillers), despite high patient demand, with most dentists rejecting the practice due to a lack of knowledge and experience. International surveys echo these barriers: in Thailand, nearly 80% of dentists supported the use of BoNT-A, but clinical uptake was constrained by inadequate training and regulatory uncertainty [12]; and in India, Pagidimarry et al. [13] reported favorable attitudes but similarly low practice rates, citing the same barrier.

In this study, patient demand was strongly associated with provision (p < 0.001). No age or gender effect was found, suggesting uptake is shaped more by systemic factors than demographics. The potential association between professional experience and the adoption of aesthetic procedures is an important factor. Dentists with more years in practice may have different patient bases, clinical habits, and perceptions of demand compared to those earlier in their careers. In this study, age was used as a proxy for experience, acknowledging that this is an indirect measure and may not fully reflect variations in years of practice. Collectively, these findings suggest that systemic enablers (training, legislation, and scope clarity) are more decisive than demographic factors in shaping adoption.

In addition, knowledge of legislation was significantly associated with performing aesthetic procedures (p < 0.001). Dentists aware of existing legal frameworks were more likely to incorporate aesthetic medicine into their practice, suggesting that regulatory familiarity may act as both a confidence factor and a perceived safeguard.

Conversely, a striking 76.5% of participants were unaware of any Moroccan legislation regulating aesthetic medicine performed by dentists. This aligns with the United Kingdom context described by Walker et al. [14], where regulatory ambiguity and lack of undergraduate training have limited integration.

Overall, this study provides the first national insight into Moroccan dentists’ attitudes, practice patterns, and awareness regarding aesthetic medicine. It highlights both latent potential through training-contingent adopters and structural barriers, such as the absence of formalized scope and training pathways. These findings support the need for regulatory clarity, targeted professional education, and further research exploring patient outcomes, procedure profiles, and long-term safety.

## Conclusions

This study reveals a growing interest in aesthetic medicine among Moroccan dentists, particularly younger practitioners and those working in private clinics. Although only a minority currently perform procedures such as BoNT-A and dermal fillers, both patient demand and dentists’ willingness suggest significant potential for expansion if appropriate training and legal clarity are established. The findings underscore the dual cosmetic and therapeutic role of these procedures and highlight dentists’ unique expertise in facial anatomy as an advantage for safe practice. Key barriers remain, including limited awareness of national regulations and insufficient training opportunities. Limitations of this study include the use of convenience sampling, the lack of data on years of professional practice, the focus on private clinics, and the use of predefined age groups. Despite these constraints, the study offers valuable insights into the evolving integration of aesthetic medicine into Moroccan dentistry, highlighting the need for structured guidelines and educational initiatives to support its safe and effective adoption.

## Appendices

**Table 7:** Questionnaire

Gender
Female
Male
Age
27-35 years
35-50 years
>50 years
Region
Casablanca-Settat
Fès-Boulemane
Marrakech-Safi
Rabat-Salé-Zemmour-Zaër
Tangier-Tétouan-Al Hoceïma
Other regions
Do you receive requests from your patients for aesthetic medical procedures?
Yes
No
In your daily practice, do you perform aesthetic medical procedures?
Yes
No
Why don’t you practice aesthetic medicine in your clinic?
I am not trained
I plan to do it after receiving training
I prefer to limit myself to the practice of dental medicine
I have no patient demand
Should dentists perform aesthetic medicine?
Yes
No
Do you have any knowledge of Moroccan legislation regarding the practice of aesthetic medicine?
Yes
No
